# Neighborhood Characteristics and Colorectal Cancer Survivors' Quality of Care

**DOI:** 10.1089/heq.2019.0062

**Published:** 2019-12-13

**Authors:** Ulrike Boehmer, Jennifer Potter, Melissa A. Clark, Al Ozonoff, Rachel M. Ceballos, Michael Winter, Kevan L. Hartshorn

**Affiliations:** ^1^ Department of Community Health Sciences, Boston University School of Public Health, Boston, Massachusetts.; ^2^ Department of Medicine, Beth Israel Deaconess Medical Center, Boston, Massachusetts.; ^3^ Harvard Medical School, Boston, Massachusetts.; ^4^ The Fenway Institute, Boston, Massachusetts.; ^5^ Department of Population and Quantitative Sciences, University of Massachusetts Medical School, Worcester, Massachusetts.; ^6^ Boston Children's Hospital, Boston, Massachusetts.; ^7^ Fred Hutchinson Cancer Research Center, Seattle, Washington.; ^8^ Biostatistics and Epidemiology Data Analytics Center, Boston University School of Public Health, Boston, Massachusetts.; ^9^ Boston University School of Medicine, Boston, Massachusetts.

**Keywords:** survivorship care plan, colorectal cancer, quality of cancer care

## Abstract

**Purpose:** Quality cancer care entails receipt of a Survivorship Care Plan (SCP). The purpose of this study was to determine differences in SCP delivery by patient-level and neighborhood characteristics.

**Methods:** We obtained California cancer registry data on individuals who were diagnosed with stage I, II, or III colorectal cancer (CRC) between 2012 and 2015 and resided in predetermined geographic areas. We then mailed them a questionnaire, which queried about receipt of a SCP and its content. SCP was defined by content, as summary of cancer treatment, cancer surveillance recommendations, and/or an individualized preventive care. Using logistic regression modeling, each measure of SCP, as well as the summary measure (none vs. any), was evaluated by person-level characteristics. Subsequently, neighborhood-level characteristics were added to the model to explore their additional value.

**Results:** Overall 80% of CRC survivors received a SCP. Receipt of SCPs was associated with person-level characteristics, while neighborhood characteristics did not make an additional contribution. Young, male employed survivors and those with more recent diagnoses or later cancer stages had greater odds of receiving a SCP.

**Conclusion:** When providing SCPs, health care providers prioritize patient groups who they may perceive as vulnerable or likely to benefit from SCPs.

## Introduction

In 2006, the Institute of Medicine (IOM) report, “From Cancer Patient to Cancer Survivor,” recommended that each cancer patient receive a Survivorship Care Plan (SCP) upon completion of treatment.^1^ Ideally, a SCP should include a summary of treatments, follow-up recommendations, and individualized recommendations for maintaining health and well-being. A 2013 IOM report defines “high quality cancer care” as meeting the needs of patients with cancer by providing them with understandable information to facilitate their engagement in informed decision making from the time of diagnosis through treatment and survivorship.^2^ Consistent with this goal, the SCP is a key document thought to facilitate survivors' engagement in care and transition from active treatment to long-term cancer survivorship.

The Commission on Cancer (CoC) issued Standard 3.3, which requires accredited cancer programs to provide a SCP to patients with stage I, II, or III cancers that are treated with curative intent, and initially outlined a stepwise implementation schedule stipulating that 10% or more patients receive a SCP by 2015, 25% or more by 2016, 50% or more by 2017, and 75% or more by 2018.^3^ Due to implementation challenges, the CoC subsequently lowered the 2018 goal from 75% to 50% of patients. Divergent from this, California set its own, more ambitious, targets of 79.1% of patients to receive a SCP by 2015, an increase over the 2011 rate of 71.9%.^4^

The widespread endorsement of SCPs catalyzed observational studies and randomized interventions that examined the benefits of SCPs.^5,6^ The literature on the benefits of SCPs is equivocal. A recent review of 13 randomized studies reported negative results for one study, in that recipients of SCPs reported more symptoms and a greater emotional impact of their cancer than those who did not receive a SCP,^6^ and seven studies had null findings, indicating that survivors who were randomized to receive a SCP did not differ in outcomes from those who did not receive a SCP.^6^

Studies of SCP delivery concluded that the receipt of SCPs is linked to patient-level factors.^7^ These findings have been confirmed in recent studies, which found that younger, non-white, more educated, and higher income patients have greater odds of receiving SCPs.^8,9^ However, study findings have been inconsistent,^8,9^ and the survivors in these studies had diverse cancer types and were geographically dispersed. This may bias findings in that cancer type and gender cannot be disaggregated, and geographic variations in SCP receipt may impact findings. To address the equitability of SCP delivery, we focused only on colorectal cancer (CRC) survivors in the state of California.

In recent years, an increasing number of studies have focused on the link between location and health, identifying associations yet also variation in the magnitude and direction of the effect.^10^ A review of studies with multilevel analyses of cancer outcomes concluded that census tracts were the most commonly used geographic scale, cancer screening the most frequent cancer outcome, and 48% of studies focused on breast cancer, while 25% focused on CRC.^11^ Prior studies of CRC in California have shown that individuals living in lower income neighborhoods have worse survival than those in higher income neighborhoods, while subsequent analyses showed that the link between neighborhood poverty and shorter survival was mediated by health insurance for women, but not men.^12,13^ Furthermore, when focusing on CRC quality of care, defined as number of regional lymph nodes examined and wait times for surgery and chemotherapy, living in poor neighborhoods was linked to worse cancer care.^12,13^ This study builds on this line of research, by performing multilevel analyses to identify which person-level and neighborhood factors best explain quality of care, defined as the receipt of a SCP among California CRC survivors.

## Methods

All aspects of the study were approved by both the Boston University and the California Department of Public Health Institutional Review Boards.

### Study setting

Eligible cases were from the California state cancer registry and had a diagnosis of colon or rectal adenocarcinoma in years 2012–2015; stage I, II, or III at diagnosis; and age 21 and older at diagnosis. We then reduced the volume of cases, by restricting ourselves to geographic areas, that is, county subdivisions, with high numbers of CRC cases and same-sex partnered households to meet the aims of a larger parent study, which sought to examine variation in CRC survivors' needs by sexual orientation.

### Recruitment process and data collection

After we received data on all potentially eligible cases from the California cancer registry for the selected geographic regions, we began contact procedures. The first contact consisted of a mailed package that entailed a brochure from the California registry explaining the function of the registry, a consent form, a study recruitment letter, a short survey, and a self-addressed stamped return envelope. The recruitment letter explained the purpose of the study, provided information about the means to opt out of the study, and announced that a member of the study team would call individuals who did not opt out of the study and did not return the questionnaire to conduct the screening survey through the telephone. A few weeks after sending the mailing, we initiated the first of a maximum of 10 call attempts, including three voice mail messages, to complete the screening survey. Between October 2015 and December 2016, we mailed study packages to 6978 patients. Of these 1040 opted out or refused participation upon contact, 429 had a language barrier, 106 were deceased, 77 had a cognitive impairment, 17 had a hearing impairment, and we were unable to make contact with 2786 due to bad addresses, nonworking phone numbers, or having made the maximum number of call attempts. We obtained 2523 completed questionnaires. We excluded 159 individuals who reported never having had cancer and one individual who was ineligible due to age, which resulted in a sample of 2363 California CRC survivors.

### Measures

All clinical and selected demographic data were obtained from the cancer registry, which cancer registrars derived from patients' medical records. Clinical information consisted of date of diagnosis, cancer site, stage at diagnosis, and number of excised lymph nodes. Treatment data entailed receipt of surgery, chemotherapy, and or radiation therapy, which we summarized into a variable that distinguished surgery only treatment from more extensive treatments. In this sample, 40 cases had multiple primary tumors, which were reviewed by the oncologist on our study to then assign appropriate clinical characteristics for each case with multiple tumors. We used the earliest diagnosis date, retained both primary sites if applicable, assigned the worst recorded cancer stage, and retained all treatments that were received. For each cancer case, we obtained an identifier for the facility at which the diagnosis and first treatment occurred from the cancer registry. We then linked the facility to the hospital annual utilization data, which are available from the California's Office of Statewide Health Planning and Development to derive a variable that distinguished teaching hospitals from nonteaching hospitals and size of the facility. Size of the facility was determined by the licensed number of medical and surgical beds and was categorized into tertiles: small (16–157 beds), medium (160–261), and large (263–591). From cancer registry demographic data, we obtained age at diagnosis; race/ethnicity categorized into non-Hispanic White, non-Hispanic Black, Hispanic, Asian/Pacific Islander, and other/unknown; and marital status, categorized as married, never married, separated, divorced, or widowed, and unknown status. From collected survey data, we obtained employment status.

From individuals' census tract at diagnosis, we derived neighborhood level descriptors of socioeconomic status. The first was the percent of the population below the poverty level. Two others captured spatial social and economic polarization, summarized as an index of concentration at the extremes (ICE) for income and race/ethnicity.^14,15^ The ICE measures range from −1, which captures the most disadvantaged, to 1, which reflects the most advantaged, while a value of 0 indicates an equal number of persons in the best off and worst off categories.^15^

Our outcome, SCP, was assessed using questions from the National Health Interview Survey with yes/no response options^7^: (1) receipt of a written summary of received treatments, which we refer to as treatment SCP; (2) receipt of written instructions of who to see for follow-up care after the completion of treatment, which we refer to as follow-up SCP; and (3) receipt of individualized recommendations for a healthy lifestyle, such as exercising and not-smoking, which we refer to as individualized SCP. Individuals who responded not knowing whether they received a SCP were counted as not having received a SCP. To provide an overall estimate of SCP receipt, we created a composite measure of SCP types 1–3 (none vs. any SCP).

### Statistical analysis

We examined the characteristics of the sample using descriptive statistics, including proportions for categorical variables and means, standard deviations, medians, and interquartile ranges for continuous variables. For each outcome variable, we used stepwise logistic regression analyses to fit a base model, including the following independent variables: sex, age at diagnosis, years since diagnosis, primary site, stage, treatment summary, number of nodes examined, race/ethnicity, marital status, and employment. Inclusion and exclusion of independent variables in the model were determined by Akaike information criterion (AIC) to determine best model fit.^16^ Each geographic descriptor was then added one by one to the best fitting base model, and model fit based on AIC criteria was examined and compared to AIC of the base model. All analyses were performed using SAS/STAT software, version 9.4 of the SAS System for Microsoft Windows.

## Results

The sample of California cancer survivors consisted of 44% men and 56% women, three-quarters were Non-Hispanic White, the majority was married, and about half of the sample self-reported as retired (Table 1). The average age at diagnosis was 63 years, with the majority diagnosed after age 50. The majority of survivors had a diagnosis of colon cancer, about 2% had multiple tumors, and cancer stage was equally distributed between stages I, II, and III. Almost 60% of survivors were treated with surgery only, while 26% were treated with chemotherapy, and 15% had chemotherapy and radiation treatment. Study contact with survivors occurred on average just before they were 3 years postdiagnosis.

**Table 1.** Characteristics of California Colorectal Cancer Survivors (*N*=2363)

	Overall	Men	Women
	*N*=2363	*N*=1039 (44%)	*N*=1324 (56%)
	*N* (%)	*N* (%)	*N* (%)
Demographics			
Race (registry)			
Non-Hispanic White	1828 (77.4)	803 (77.3)	1025 (77.4)
Non-Hispanic Black	182 (7.7)	77 (7.4)	105 (7.9)
Hispanic	120 (5.1)	46 (4.4)	74 (5.6)
Asian/Pacific Islander	201 (8.5)	105 (10.1)	96 (7.3)
American Indian/other/unknown	32 (1.4)	8 (0.8)	24 (1.8)
Marital status (registry)			
Married	1272 (57.1)	634 (63.9)	638 (51.6)
Separated/divorced/widowed	481 (21.6)	123 (12.4)	358 (28.9)
Single, never married	476 (21.4)	235 (23.7)	241 (19.5)
Missing	134	47	87
Employment (screener)			
Employed	873 (37.4)	447 (43.6)	426 (32.6)
Looking for work/other	94 (4.0)	30 (2.9)	64 (4.9)
Retired	1157 (49.6)	463 (45.1)	694 (53.1)
On disability/unable to work	209 (9.0)	86 (8.4)	123 (9.4)
Missing	30	13	17
Clinical characteristics			
Age at diagnosis			
Range	22–97	23–94	22–97
Median (IQR)	65 (55, 72)	64 (55, 72)	65 (56, 73)
Mean (SD)	63.4 (11.8)	62.9 (11.7)	63.8 (11.8)
21–49 years old	288 (12.2)	134 (12.9)	154 (11.6)
50–64 years old	882 (37.3)	408 (39.3)	474 (35.8)
65 years and older	1193 (50.5)	497 (47.8)	696 (52.6)
Primary site			
Colon	1621 (68.6)	672 (64.7)	949 (71.7)
Rectum	731 (30.9)	358 (34.5)	373 (28.2)
Both	11 (0.5)	9 (0.9)	2 (0.1)
Multiple tumors	39 (1.7)	23 (2.2)	16 (1.3)
Stage at diagnosis			
Stage I	746 (31.6)	335 (32.2)	411 (31.0)
Stage II	726 (30.7)	306 (29.5)	420 (31.7)
Stage III	891 (37.7)	398 (38.3)	493 (37.2)
Regional nodes examined			
None	221 (9.4)	111 (10.8)	110 (8.3)
1–5	71 (3.0)	34 (3.3)	37 (2.8)
6–10	142 (6.0)	58 (5.6)	84 (6.4)
11–14	522 (17.9)	186 (18.0)	236 (17.8)
15+	1499 (63.7)	643 (62.3)	856 (64.7)
Unknown/missing	8	7	1
Treatment summary			
Surgery only	1309 (55.4)	553 (53.2)	756 (57.1)
Chemotherapy/no radiation	620 (26.2)	273 (26.3)	347 (26.2)
Chemotherapy+radiation	347 (14.7)	172 (16.5)	175 (13.2)
Other/unknown	87 (3.7)	41 (4.0)	46 (3.5)
Time since diagnosis (years)			
Range	1.1–4.6	1.1–4.6	1.1–4.6
Median (IQR)	2.8 (1.9, 3.5)	2.8 (1.8, 3.5)	2.9 (2.0, 3.5)
Mean (SD)	2.8 (0.9)	2.7 (0.9)	2.8 (0.9)
Structural characteristics			
ICE for race			
Range	−0.77–1.00	−0.77–1.00	−0.77–0.96
Median (IQR)	0.59 (0.33, 0.75)	0.57 (0.31, 0.74)	0.59 (0.33, 0.75)
Mean (SD)	0.50 (0.31)	0.50 (0.31)	0.51 (0.31)
ICE for income			
Range	−1.00–0.84	−1.00–0.84	−0.72–0.84
Median (IQR)	0.17 (−0.03, 0.36)	0.18 (−0.02, 0.37)	0.15 (−0.04, 0.36)
Mean (SD)	0.16 (0.28)	0.17 (0.28)	0.15 (0.27)
Percent below poverty level			
Range	0–80.3	0–80.3	0.1–62.7
Median (IQR)	9.4 (5.4, 15.9)	9.5 (5.5, 16.0)	9.2 (5.3, 15.9)
Mean (SD)	12.0 (9.2)	12.0 (9.2)	11.9 (9.2)
Teaching hospital			
Yes	540 (25.1)	273 (29.1)	267 (22.1)
No	1608 (74.9)	666 (70.9)	942 (77.9)
Missing	215	100	115
Hospital size			
Low	717 (33.4)	252 (26.8)	465 (38.5)
Medium	695 (32.4)	320 (34.1)	375 (31.0)
High	736 (34.3)	367 (39.1)	369 (30.5)
Missing	215	100	115

ICE, index of concentration at the extremes; IQR, interquartile range; SD, standard deviation.

The mean value of the ICE for race indicated that survivors lived in areas with a greater number of white residents, and the ICE for income showed that these areas were financially better off. Survivors lived in areas in which on average 12% of residents lived below the poverty level. One quarter of survivors received their diagnosis and first course of treatment at teaching hospitals with cases distributed across facilities of all sizes.

Twenty percent of survivors reported receiving no SCP irrespective of type. Among those receiving an SCP, the percentage of those receiving various types of SCP was relatively consistent, with 50% reporting receipt of a treatment summary, 56% a follow-up SCP, and 56% an individualized SCP.

The base model in Table 2 summarizes individual-level explanatory factors that best explain the receipt of a treatment SCP. Younger, male, black or Hispanic, more recently diagnosed survivors, and those with a later stage of cancer at the time of diagnosis had a greater likelihood of receiving a treatment SCP than older, female, non-Hispanic White survivors with greater time elapsed since diagnosis and those with earlier stage disease. Adding any of the three neighborhood characteristics to this best fitting model composed of individual factors did not improve model fit for receipt of a treatment plan. Furthermore, none of the geographic factors changed any of the individual factors in the strength or direction of the association.

**Table 2.** Explanatory Factors for Receipt of a Written Summary of Received Treatments (Treatment Survivorship Care Plan)

	Base model	Adding ICE race	Adding ICE income	Adding poverty level
	OR (95% CI)	OR (95% CI)	OR (95% CI)	OR (95% CI)
Sex				
Male	1.15 (0.98–1.36)	1.15 (0.98–1.36)	1.15 (0.98–1.36)	1.15 (0.98–1.36)
Female	REF	REF	REF	REF
Race				
White	REF	REF	REF	REF
Black	1.46 (1.07–1.99)	1.54 (1.09–2.18)	1.47 (1.07–2.01)	1.46 (1.06–2.01)
Hispanic	1.93 (1.30–2.88)	1.98 (1.32–2.97)	1.94 (1.30–2.89)	1.93 (1.29–2.88)
Asian/Pacific Islander	1.19 (0.88–1.60)	1.22 (0.90–1.67)	1.19 (0.88–1.60)	1.19 (0.88–1.60)
Other/unknown	1.60 (0.78–3.27)	1.63 (0.80–3.35)	1.60 (0.78–3.28)	1.59 (0.78–3.27)
Age at diagnosis				
21–49	1.27 (0.97–1.67)	1.27 (0.97–1.67)	1.27 (0.97–1.67)	1.27 (0.97–1.67)
50–64	REF	REF	REF	REF
65+	0.85 (0.71–1.01)	0.85 (0.71–1.01)	0.85 (0.71–1.01)	0.85 (0.71–1.01)
Years since diagnosis	0.88 (0.80–0.96)	0.88 (0.80–0.97)	0.88 (0.80–0.97)	0.88 (0.80–0.97)
Stage				
I	REF	REF	REF	REF
II	0.97 (0.79–1.19)	0.97 (0.79–1.19)	0.97 (0.79–1.19)	0.97 (0.78–1.19)
III	1.14 (0.93–1.38)	1.13 (0.93–1.38)	1.14 (0.93–1.38)	1.14 (0.93–1.38)
ICE race		1.12 (0.82–1.52)		
ICE income			1.01 (0.74–1.38)	
Poverty				1.00 (0.99–1.01)
Model AIC	3216.702	3218.194	3218.695	3218.698

AIC, Akaike information criterion; CI, confidence interval; OR, odds ratio; REF, reference group.

Survivors who were not married, older at diagnosis, or looking for/unable to work were less likely to receive a follow-up SCP than those who were married, diagnosed between ages 50 and 64, or employed, while survivors with more nodes examined and receiving more extensive treatments had a greater likelihood of receiving a follow-up SCP than survivors with fewer nodes or those treated with surgery only (Table 3). None of the neighborhood characteristics changed the individual level factors in magnitude or direction and did not improve the best fitting explanatory model of follow-up SCP.

**Table 3.** Explanatory Factors for Receipt of Written Instructions of Who to See for Follow-Up Care After the Completion of Treatment (Follow-Up Survivorship Care Plan)

	Base model	Adding ICE race	Adding ICE income	Adding poverty level
	OR (95% CI)	OR (95% CI)	OR (95% CI)	OR (95% CI)
Marital status				
Single	0.78 (0.63–0.97)	0.77 (0.62–0.96)	0.79 (0.63–0.99)	0.78 (0.62–0.97)
Married	REF	REF	REF	REF
Separated/divorced/widowed	0.79 (0.63–0.98)	0.77 (0.62–0.96)	0.79 (0.64–0.99)	0.78 (0.63–0.98)
Employment				
Employed	REF	REF	REF	REF
Looking for work	0.65 (0.41–1.00)	0.64 (0.41–1.00)	0.65 (0.41–1.01)	0.64 (0.41–1.00)
Retired	1.00 (0.79–1.27)	0.99 (0.78–1.27)	1.00 (0.79–1.27)	1.00 (0.79–1.27)
Unable	0.62 (0.45–0.85)	0.61 (0.44–0.84)	0.63 (0.46–0.86)	0.62 (0.45–0.85)
Age at diagnosis				
21–49	0.97 (0.72–1.29)	0.97 (0.72–1.29)	0.96 (0.72–1.29)	0.97 (0.72–1.30)
50–64	REF	REF	REF	REF
65+	0.70 (0.55–0.88)	0.71 (0.56–0.89)	0.70 (0.55–0.88)	0.70 (0.56–0.89)
Nodes examined				
0–5	REF	REF	REF	REF
6+	1.27 (0.97–1.67)	1.27 (0.97–1.67)	1.27 (0.97–1.67)	1.27 (0.97–1.67)
Treatment summary				
Chemotherapy/no radiation	1.32 (1.07–1.63)	1.33 (1.08–1.64)	1.32 (1.07–1.63)	1.32 (1.07–1.63)
Chemotherapy+radiation	1.29 (0.99–1.67)	1.30 (1.00–1.88)	1.28 (0.99–1.67)	1.29 (0.99–1.67)
Surgery only	REF	REF	REF	REF
Other/unknown	1.45 (0.89–2.35)	1.44 (0.89–2.34)	1.46 (0.90–2.37)	1.45 (0.89–2.35)
ICE race		0.83 (0.63–1.10)		
ICE income			1.11 (0.81–1.53)	
Poverty				1.00 (0.99–1.01)
Model AIC	2948.245	2948.601	2949.826	2950.115

Male survivors with more recent diagnoses, more examined nodes, and more extensive cancer treatment were more likely to receive an individualized SCP than women or those who were diagnosed more distantly, had fewer examined nodes, or were treated with surgery only (Table 4). None of the neighborhood characteristics improved the explanation of receipt of an individualized SCP.

**Table 4.** Explanatory Factors for Receipt of Individualized Recommendations for a Healthy Lifestyle (Individualized Survivorship Care Plan)

	Base model	Adding ICE race	Adding ICE income	Adding poverty level
	OR (95% CI)	OR (95% CI)	OR (95% CI)	OR (95% CI)
Sex				
Male	1.21 (1.02–1.43)	1.21 (1.02–1.43)	1.21 (1.02–1.43)	1.21 (1.02–1.43)
Female	REF	REF	REF	REF
Years since diagnosis	0.90 (0.82–0.99)	0.90 (0.82–0.99)	0.90 (0.82–0.99)	0.90 (0.82–0.99)
Nodes examined				
0–5	REF	REF	REF	REF
6+	1.59 (1.22–2.06)	1.59 (1.23–2.06)	1.59 (1.23–2.06)	1.58 (1.22–2.05)
Treatment summary				
Chemotherapy/no radiation	1.60 (1.31–1.95)	1.60 (1.31–1.95)	1.60 (1.31–1.95)	1.60 (1.31–1.95)
Chemotherapy+radiation	1.25 (0.98–1.60)	1.26 (0.98–1.61)	1.25 (0.98–1.60)	1.25 (0.98–1.60)
Surgery only	REF	REF	REF	REF
Other/unknown	1.20 (0.77–1.88)	1.20 (0.77–1.88)	1.20 (0.77–1.88)	1.21 (0.77–1.90)
ICE race		0.92 (0.70–1.20)		
ICE income			0.99 (0.73–1.34)	
Poverty				1.00 (0.99–1.01)
Model AIC	3182.047	3183.620	3184.042	3183.568

In the Table 5 we summarize the likelihood of not receiving any SCP. The likelihood of not receiving a SCP was greatest for women, those looking for work or unable to work, aged 65 or older at the time of diagnosis, and those diagnosed with stage I cancer a longer time ago. None of the geospatial characteristics changed any of the individual explanatory factors. Of the geographic factors, none improved the best fitting explanatory model for not receiving a SCP. However, after controlling for individual level factors, ICE for income and neighborhood poverty were significantly associated with receipt of SCP, indicating that individuals in economically advantaged neighborhoods are more likely to receive a SCP.

**Table 5.** Explanatory Factors for Not Receiving Any Survivorship Care Plan

	Base model	Adding ICE race	Adding ICE income	Adding poverty level
	OR (95% CI)	OR (95% CI)	OR (95% CI)	OR (95% CI)
Sex				
Male	0.83 (0.67–1.03)	0.83 (0.67–1.03)	0.84 (0.68–1.04)	0.83 (0.67–1.03)
Female	REF	REF	REF	REF
Employment				
Employed	REF	REF	REF	REF
Looking for work	1.85 (1.10–3.09)	1.84 (1.10–3.07)	1.85 (1.11–3.10)	1.84 (1.10–3.08)
Retired	1.04 (0.77–1.39)	1.03 (0.77–1.39)	1.04 (0.77–1.39)	1.04 (0.77–1.39)
Unable	1.58 (1.07–2.33)	1.56 (1.05–2.30)	1.51 (1.02–2.24)	1.53 (1.04–2.26)
Age at diagnosis				
21–49	0.68 (0.45–1.02)	0.68 (0.45–1.02)	0.69 (0.46–1.04)	0.68 (0.45–1.03)
50–64	REF	REF	REF	REF
65+	1.57 (1.19–2.08)	1.58 (1.19–2.09)	1.58 (1.20–2.10)	1.58 (1.20–2.09)
Years since diagnosis	1.19 (1.06–1.34)	1.19 (1.06–1.34)	1.19 (1.06–1.35)	1.19 (1.06–1.34)
Stage				
I	REF	REF	REF	REF
II	0.97 (0.75–1.26)	0.97 (0.75–1.26)	0.96 (0.75–1.25)	0.97 (0.75–1.25)
III	0.79 (0.61–1.02)	0.79 (0.61–1.02)	0.79 (0.61–1.02)	0.79 (0.61–1.02)
ICE race		0.88 (0.63–1.22)		
ICE income			0.67 (0.46–0.99)	
Poverty				1.01 (1.00–1.02)
Model AIC	2249.688	2251.086	2247.616	2249.364

In separate exploratory analyses (results not shown), we added one at a time the two facility variables—teaching status and size—to the base model of each type of SCP to examine whether these variables improved model fit. Neither one improved the best fitting explanatory models for any of the SCP types, according to the AIC.

## Discussion

SCPs have been promoted as a key strategy to provide cancer survivors with understandable information to facilitate engagement in care during the transition from active treatment to long-term survivorship. Overall, 80% of California CRC survivors in this study reported receiving at least one type of SCP with specific SCP types received by a minimum of 50% of patients, outperforming targets set by the CoC.^3^ By comparison, analyses of the 2010 data from the National Health Interview Survey showed that 38.2% of individuals with any type of cancer reported receiving a treatment summary and 60.5% of cancer survivors reported receiving written advice on follow-up care.^8^ The higher rate of SCPs observed in this study is likely due to more recent collection of our study data and suggests that by 2016, SCP delivery reached California state targets in the geographic areas we studied. It is worth noting that 6.3% of survivors who completed the survey reported not having cancer, which implies that they were unaware of their cancer diagnosis and the need for dedicated follow-up—the very issues addressed by a SCP. California's cancer control plan includes health provider education and widespread efforts to integrate SCPs into systems of care as strategies to achieve positive health outcomes.^4^ From the data available to us, we cannot determine which of these strategies made it possible for California to achieve this remarkable prevalence of SCP delivery.

Despite this almost standardized delivery of SCPs, it is notable that this study confirmed prior findings of disparities in SCP delivery.^8,9^ In this study of CRC survivors, we were able to link age, gender, time since diagnosis, cancer stage, cancer treatments, number of nodes examined, employment, marital status, and race to the receipt of SCP. Thus, while we were able to show that four out of five CRC patients in California receive a SCP, the deviations we found suggest that providers may prioritize SCP delivery to CRC patients whom they perceive as more vulnerable, that is, those with more advanced cancer stage or extensive treatment, or younger patients, who may reap the most benefits from longitudinal follow-up. This is consistent with findings of an Australian study of CRC patients, which noted a pattern of “positive discrimination” in receipt of SCPs, with survivors living in remote areas, non-English speakers, and those with no experience accessing the health care system more likely to receive a SCP.^17^ Similarly, in our study, patient groups with greater difficulty accessing the health care system—those who are unemployed, racial/ethnic minority patients, and men who are known to access health care less—had a greater likelihood of receiving a SCP. Similarly, a prior study found that non-White patients were more likely to receive SCPs, hypothesizing that non-White race may be a proxy for more complex medical needs due to multiple comorbid conditions and therefore a greater likelihood of receiving a SCP.^8^ We found only two exceptions to this prioritization pattern. First, patients with a more recent diagnosis had a greater likelihood of receiving a SCP, most likely due to being in closer proximity to the time in which SCP receipt has become an expectation. Second, married survivors had a greater likelihood of receiving follow-up SCPs, possibly due to involvement of spousal caregivers in the planning process, who may take particular interest in follow-up care. The involvement of caregivers and their perspectives regarding SCPs has previously been noted as an unexplored aspect of SCP implementation.^18^

Conceptually, it has been recognized that neighborhoods are determinants of health, including determinants of cancer outcomes across the cancer continuum.^10^ We therefore examined the contribution of geographic characteristics on the quality of cancer care among California CRC survivors. Others have successfully linked neighborhood socioeconomic status (SES) or racial segregation to cancer outcomes, above and beyond the effects of individual socioeconomic status or race.^10,19^ In our data neighborhood SES did not meaningfully improve the explanatory model of receiving a SCP, despite the existence of an association between neighborhood SES and receipt of SCPs, after individual level factors were controlled. We suggest that cancer outcomes impacted by neighborhood characteristics tend to depend on patient behaviors, such as engaging in healthy behaviors or cultural perceptions of treatments,^20^ whereas the delivery of SCP is dependent on physician behaviors. Future studies that use multilevel modeling of SCP delivery should consider factors that affect physician behaviors, such as hospital or practice characteristics.

Despite this study's many strengths, we acknowledge a number of limitations. Our study lacked health insurance data, which would allow for a further determination of differences in SCP delivery. In addition, patients had to be English speaking to be eligible for this study, which prevented us from addressing linguistic differences in SCP receipt. Because our data on facility characteristics reflected only facilities at diagnosis and for the first course of treatment, we consider our investigation of possible links between facility characteristics and SCP to be exploratory. Our selection of geographic areas with a high number of CRC cases and high numbers of same-sex partnered households is a potential source of bias. While we did not measure actual SCP content or query patients about the perceived usefulness of the information they received, our study's assessment of specific types of SCP is a strength of our study. Furthermore, because we obtained acknowledgment of receipt of SCP from survivors directly, our findings may have greater validity than electronic medical record assessments alone, which can document that SCPs are generated yet fail to elucidate how often they are actually delivered to survivors.

Overall, the high rate of SCPs received by California CRC survivors points to successful implementation of quality improvement in cancer care. However, even in a state with a largely successful distribution of SCPs, important differences persist, which may stem from providers' prioritization of patients perceived to have the greatest need or benefit. These findings suggest opportunities to improve survivorship care further by eliminating disparities in SCP delivery so that all survivors can benefit.

## Abbreviations Used

AIC: Akaike information criterion
CI: confidence interval
CoC: commission on cancer
CRC: colorectal cancer
ICE: index of concentration at the extremes
IOM: Institute of Medicine
IQR: interquartile range
OR: odds ratio
SCP: survivorship care plan
SD: standard deviation
SES: socioeconomic status
